# Supplementary material for: Harnessing heterogeneous nucleation to control tin orientations in electronic interconnections
Source: Nat Commun. 2017 Dec 4;8:1916. doi: 10.1038/s41467-017-01727-6 (PMC5715011; doi:10.1038/s41467-017-01727-6)
Supplement: Supplementary file 1 — Supplementary Information [file 41467_2017_1727_MOESM1_ESM.pdf]

Supplementary Information

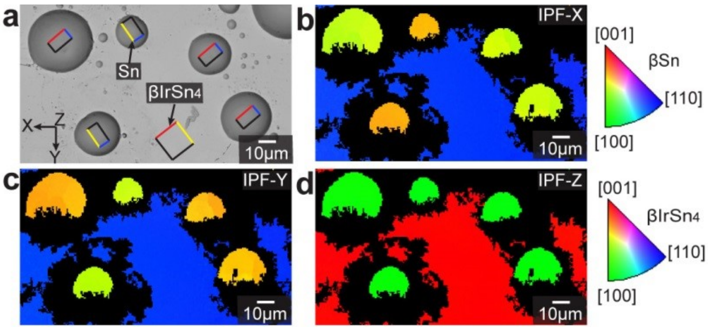

Supplementary Figure 1 A typical example of Sn droplets solidified on the (001) facet of βIrSn4 and the corresponding EBSD maps. a The backscatter electron image. **b** IPF-X map. **c** IPF-Y map. **d** IPF-Z map. This is an expanded version of Figure 3d.

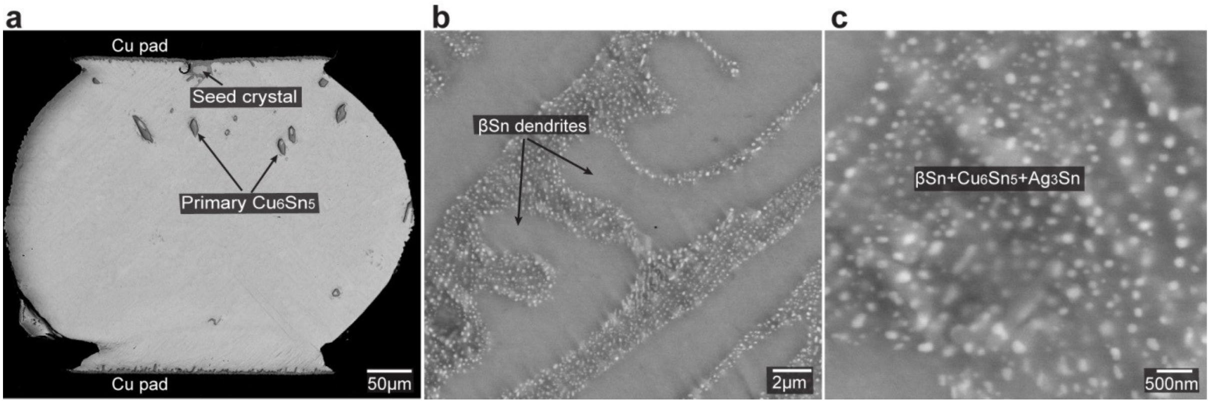

Supplementary Figure 2 Microstructure of a typical Cu/Sn-3Ag-0.5Cu or Sn-3.5Ag + IMC/Cu solder joint. a The macrostructure of a joint in which the seed crystal and numerous primary Cu_6Sn_5 phases can be seen. There are no primary Ag_3Sn phases. The microstructures shown **b** βSn dendrites and **c** βSn + Cu_6Sn_5 + Ag_3Sn ternary eutectic.

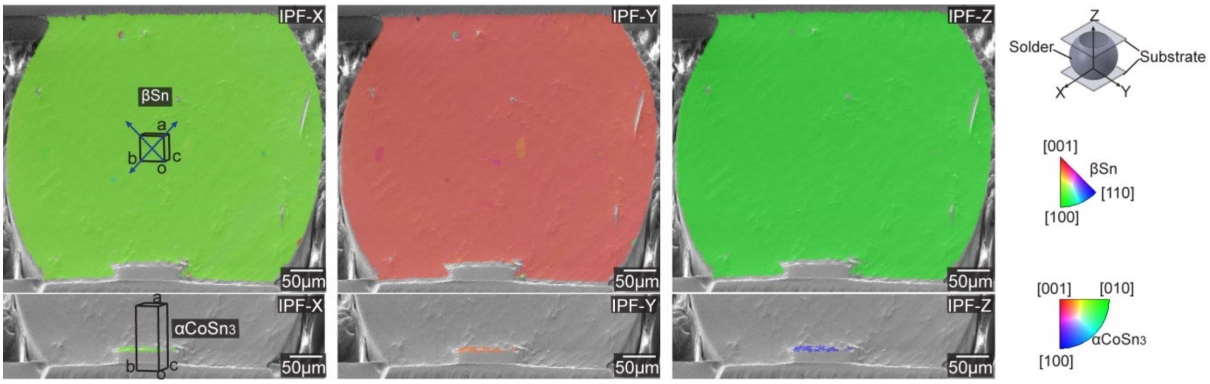

Supplementary Figure 3 An expanded version of Figure 5d showing EBSD maps

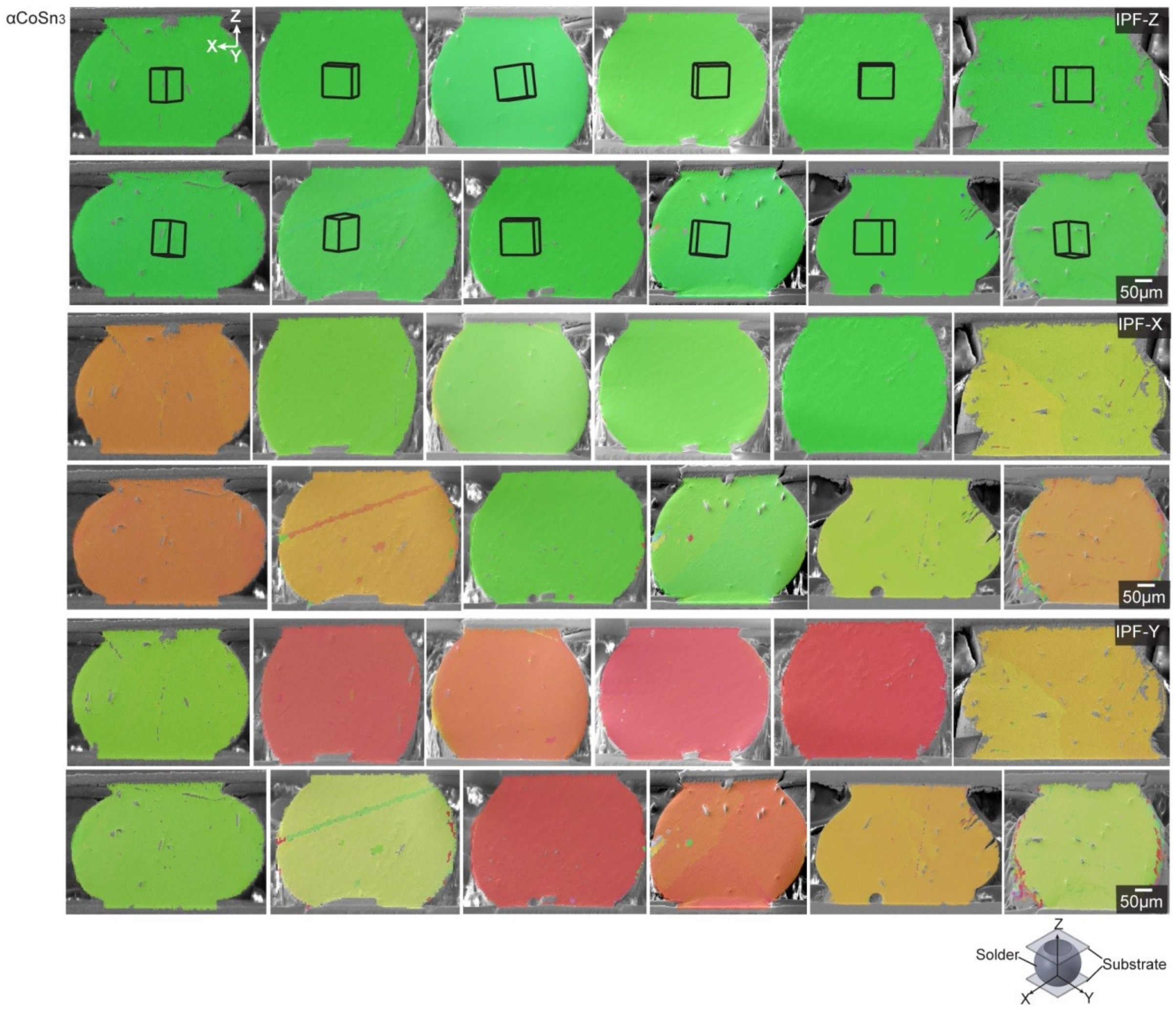

Supplementary Figure 4 An expanded version of Figure 6a showing EBSD maps. Note that the mechanical twin across one sample and minor orientations at the sample edges were caused by deformation during sample preparation.

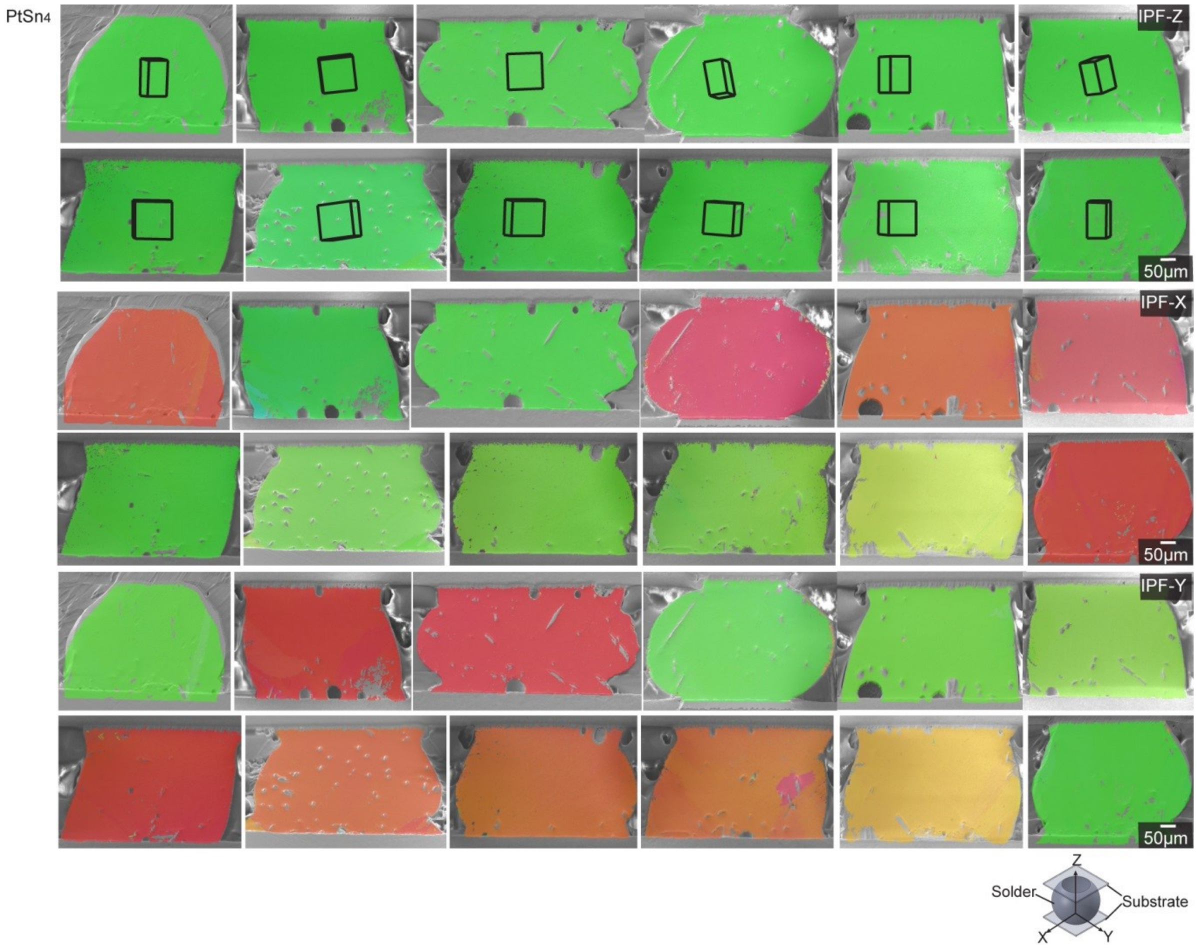

Supplementary Figure 5 An expanded version of Figure 6b showing EBSD maps.

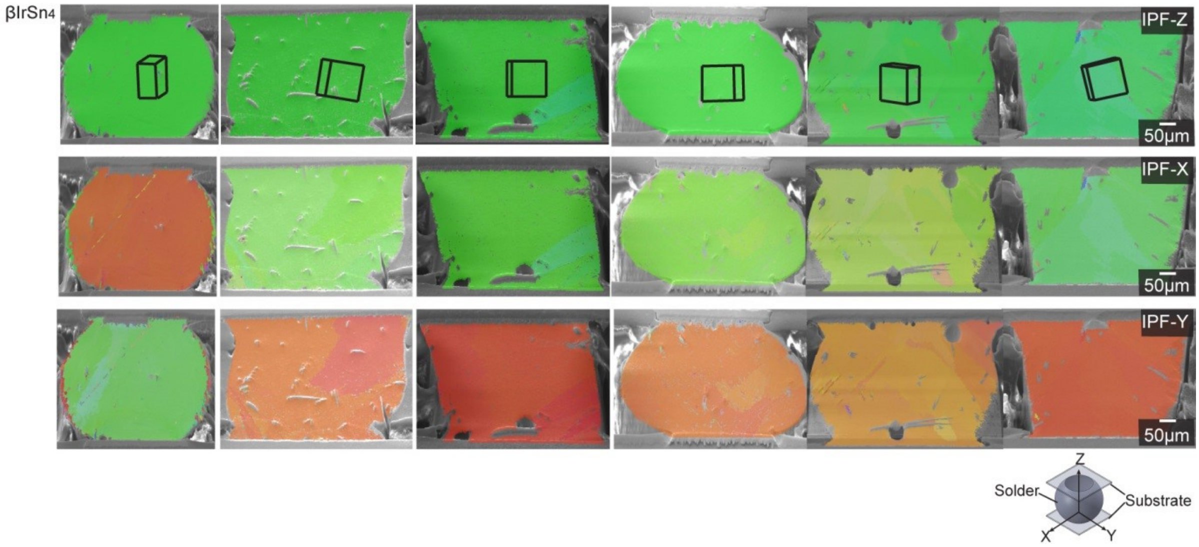

Supplementary Figure 6 An expanded version of Figure 6c showing EBSD maps .

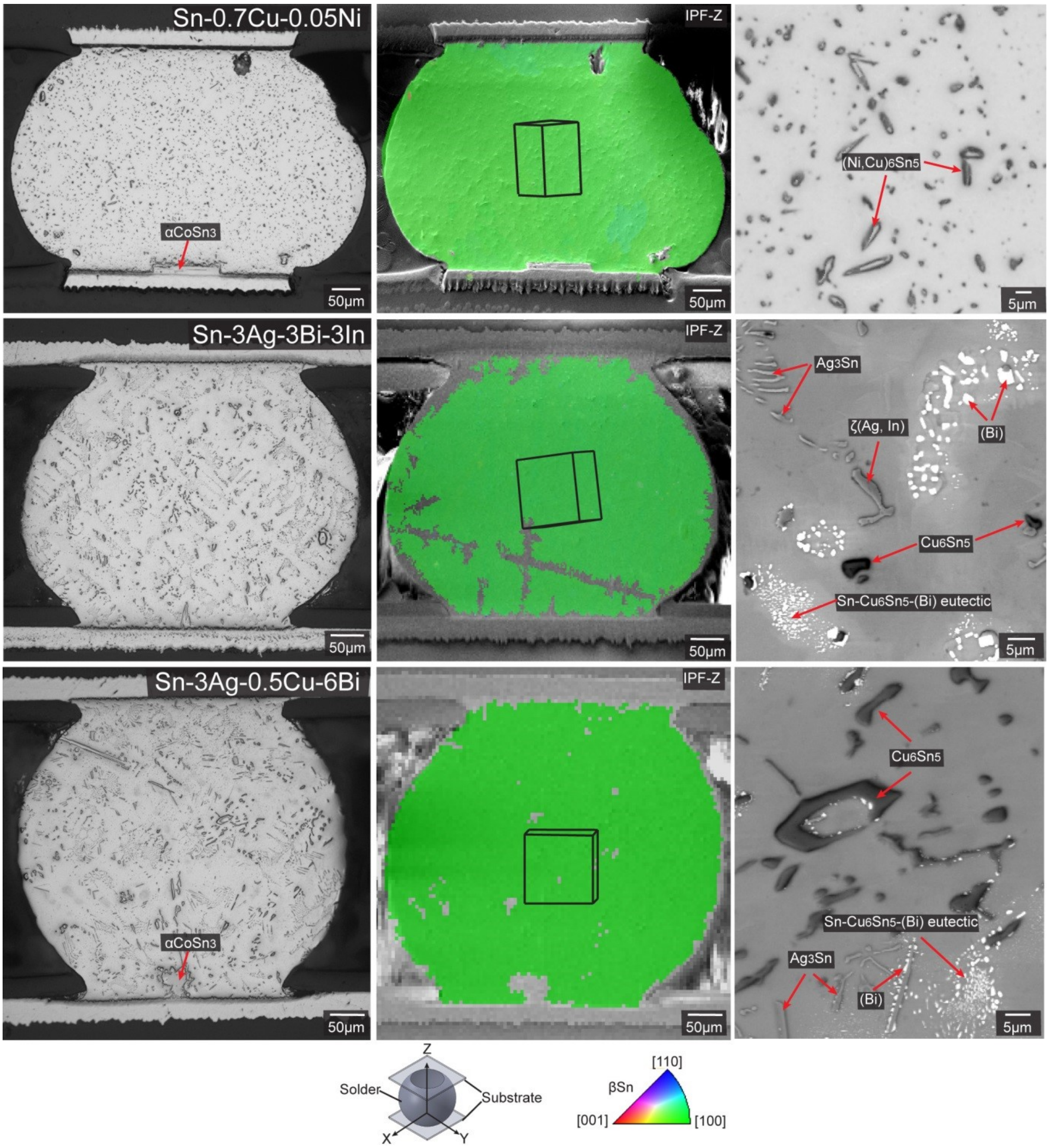

Supplementary Figure 7 c-axis orientation control in joints of different alloys. The nucleant IMCs used are α$CoSn_3$. From left to right, optical micrographs, EBSD IPF-Z maps, and micrographs shown phases in solder joints.

Supplementary Table 1 Compositions of lab-made alloys measured by XRF spectroscopy

Alloys	Ag [wt%]	Cu [wt%]	Co [wt%]	Pb [wt%]	Sb [wt%]	Bi [wt%]	Ni [wt%]	Fe [wt%]	Zn [wt%]	In [wt%]	Sn [wt%]
SAC305	3.200	0.470	<0.001	0.006	0.018	<0.001	0.002	<0.001	<0.001	0.002	Bal.
Sn-3.5Ag	3.300	0.006	0.001	0.008	0.019	<0.001	0.003	0.003	<0.001	0.009	Bal.
Sn-3Ag-3Bi-3In	2.830	0.005	<0.001	0.020	<0.001	2.990	0.004	0.006	<0.001	3.150	Bal.
Sn-3Ag-0.5Cu-6Bi	2.540	0.512	<0.001	0.009	0.016	6.080	0.006	0.006	<0.001	<0.001	Bal.
